# Future Proofing Study: a cluster randomised controlled trial evaluating the effectiveness of a universal school-based cognitive–behavioural programme for adolescent depression

**DOI:** 10.1136/bmjment-2024-301426

**Published:** 2025-03-14

**Authors:** Aliza Werner-Seidler, Andrew Mackinnon, Philip J Batterham, Alison L Calear, Mark E Larsen, Michelle Torok, Bridianne O’Dea, Kate Maston, Kit Huckvale, Hiroko Fujimoto, Lara Johnston, Lyndsay Brown, Alexandra Batholomew, Debopriyo Bal, Joanne R Beames, Susan Rachel Skinner, Katherine M Boydell, Susanne Schweizer, Raghu Lingam, Yael Perry, Jennifer L Hudson, Ju Lee Oei, Katharine Steinbeck, Maree Teesson, Svetha Venkatesh, Helen Christensen

**Affiliations:** 1 Black Dog Institute, UNSW Sydney, Sydney, New South Wales, Australia; 2 School of Psychology, University of New South Wales, Sydney, New South Wales, Australia; 3 Centre for Mental Health Research, Australian National University, Canberra, Australian Capital Territory, Australia; 4 Centre for Big Data Research in Health, University of New South Wales, Sydney, New South Wales, Australia; 5 Institute for Mental Health and Wellbeing, Flinders University, Adelaide, South Australia, Australia; 6 Centre for Digital Transformation of Health, University of Melbourne, Melbourne, Victoria, Australia; 7 Center for Contextual Psychiatry, Department of Neurosciences, KU Leuven, Leuven, Belgium; 8 Discipline of Child and Adolescent Health, Faculty of Medicine and Health, The University of Sydney, Sydney, New South Wales, Australia; 9 School of Women’s and Children’s Health, University of New South Wales, Sydney, New South Wales, Australia; 10 The Kids Research Institute, The University of Western Australia, Perth, Western Australia, Australia; 11 Discipline of Psychiatry and Mental Health, University of New South Wales, Sydney, New South Wales, Australia; 12 The Matilda Centre for Research in Mental Health and Substance Use, The University of Sydney, Sydney, New South Wales, Australia; 13 Applied Artificial Intelligence Institute, Deakin University, Melbourne, Victoria, Australia

**Keywords:** Child & adolescent psychiatry, Depression & mood disorders

## Abstract

**Background:**

Psychological prevention programmes delivered in schools may reduce symptoms of depression. However, high-quality, large-scale trials are lacking.

**Objective:**

The aim was to examine whether a digital cognitive–behavioural programme (‘SPARX’), delivered at scale in schools, would reduce depressive symptoms 12 months later.

**Methods:**

A cluster randomised controlled trial with parallel arms (intervention; control) was conducted in Australian schools, between August 2019 and December 2022. Cluster randomisation occurred at the school level (1:1 allocation). Investigators were blind to group allocation, and outcomes were assessed at baseline, 6 weeks, 6 months (primary outcome only) and 12 months post baseline. The intervention was delivered via smartphone app. Schools were instructed to provide in-class time for intervention completion. The primary outcome was the difference in depressive symptom change from baseline to 12 months between the intervention and control group. Secondary outcomes were change in anxiety, psychological distress and insomnia.

**Findings:**

134 schools participated in this study, and baseline data were collected from n=6388 students (n=3266 intervention; n=3122 control). Intent-to-treat analyses showed no difference in depression change between groups from baseline to 12 months, (mean change difference= −0.05, z= −0.32, 95% CI: −0.36 to 0.23, p=0.75). There were no differences on secondary outcomes. Many schools did not provide in-class time for intervention completion, and engagement was low (22% completion rate).

**Conclusions:**

Scaled delivery of a digital cognitive–behavioural programme did not reduce symptoms of depression, relative to a control group.

**Clinical implications:**

Given the variability in the engagement with and delivery of the digital universal cognitive–behavioural programme, caution is required prior to scaled delivery of SPARX in school contexts.

**Trial registration number:**

ACTRN12619000855123.

**WHAT IS ALREADY KNOWN ON THIS TOPIC**

**WHAT THIS STUDY ADDS**

- In a large randomised controlled trial with a 12-month primary outcome timepoint, this study found that a previously tested cognitive–behavioural programme (‘SPARX’), when delivered at scale, did not reduce depressive symptoms compared to a control group.

**HOW THIS STUDY MIGHT AFFECT RESEARCH, PRACTICE OR POLICY**

- This evidence should guide policy makers in their recommendations for universal school initiatives, and future research should consider school-specific implementation factors and intervention tailoring to meet student needs.

## Background

 In response to rising prevalence and disease burden, there have been calls for greater investment in research and focus on the prevention of depression in adolescence.^1 2^ Universal prevention programmes are often delivered in secondary schools because they can reach large numbers of young people, often before the age that mental health problems first develop.^3^ Meta-analytic reviews indicate small preventive effects of school-based prevention programmes on symptoms of depression and anxiety^4^ and internalising disorders overall.^5^ However, these conclusions are limited by considerable risk of bias in approximately two-thirds of studies to date, a lack of long-term follow-up data, and most notably, small sample sizes which are often associated with inadequate power.^4^ For example, the median sample size in a meta-analysis was 209 participants. Only one study of a universally delivered programme recruited more than 2000 students,^6^ which reported null results. This is consistent with two large school-based prevention trials recently published involving thousands of participants, both of which also found null effects.^7 8^ Further high-quality trials using large samples are critically needed to understand if universal prevention can be effective in school settings. These studies need to involve evidence-based programmes that are scalable, use large samples, attend to implementation processes and include long-term follow-up.

### Objective

The aim of this study was to investigate the effects of scalable, effective prevention programme for adolescent depression, relative to a control group. This study involved two stages: stage 1 was a cluster randomised universal prevention trial and stage 2, implemented 12-months post baseline, used an indicated prevention approach for participants showing elevated depressive symptoms.^9^ This paper reports on stage 1 where a gamified, self-directed digital cognitive–behavioural programme, ’SPARX’,^10^ was delivered universally. Our team previously found SPARX to be effective in reducing depressive symptoms in a universal sample of senior secondary school students.^11^ In the current trial, the target sample consisted of students aged 13–14 years. This age was specifically selected on the basis that symptoms of depression increase rapidly from this time, making it an optimal time for prevention.^12^ It was hypothesised that students from schools allocated to the intervention would show lower levels of depressive symptoms, the primary outcome, at 12 months post baseline relative to students from schools in the control condition. Beneficial effects in the intervention group relative to the control group on other mental health-related secondary outcomes were also expected.

## Methods

This study was prospectively registered on the Australian and New Zealand Clinical Trial Registry (ACTRN12619000855123), Consolidated Standards of Reporting Trials guidelines followed,^13^ a protocol paper,^9^ process evaluation^14^ and baseline characteristics paper^15^ published. This trial was underway during the COVID-19 pandemic, and adjustments to data collection methods were necessary during October 2020 and July–September 2021, where some assessments were conducted remotely via Zoom when students were learning from home because of lockdowns. This impacted 1% of participants, of which 46% were in the intervention group and 54% in the control group.

### Study design and participants

A cluster randomised, controlled, single-blind trial that consisted of two parallel arms (intervention and control) with 1:1 allocation was conducted. A cluster approach was selected to avoid potential contamination within schools. Recruitment occurred between March 2019 and March 2022. All schools located in the state of New South Wales (Australia) were invited to participate via email invitation. Non-government schools in capital cities around Australia were also invited to participate. A member of the research team contacted invited schools with a phone call to explore interest in participation and explain the study details. For eligibility, schools were required to have a counsellor or well-being staff member onsite during the data collection visits. Year 8 (13–14 years) students at participating schools were invited to take part. Eligibility criteria included participants attending a participating school, ability to provide active informed consent from both the parent/guardian and student, a mobile phone number and a smartphone with iOS or Android operating system. There were no restrictions placed on whether participants could seek or change treatments or therapy during the study period. There were no exclusion criteria.

### Randomisation and blinding

Schools were randomised with a 1:1 allocation using a computer-generated randomisation schedule, stratified by school size (smaller/larger than 400 students), school location (metropolitan vs regional), school type (coeducational or gender selective) and socioeconomic level (Index of Community Socio-Educational Advantage^16^; high vs low). The trial statistician conducted the randomisation and was unaware of school identity (and hence condition) throughout the trial and analyses. School allocation was communicated only to the trial manager and team running the trial day to day. The investigator team were unaware of allocation. Schools were not explicitly informed of their allocation but could deduce it from study activities. All outcome assessments were conducted electronically and therefore not subject to assessor bias.

### Outcomes and data collection methods

Study outcomes were assessed on student laptops in class and occurred at baseline, post intervention (6 weeks post baseline) and follow-up (12 months post baseline; primary endpoint). The assessments were facilitated by our research team who attended schools in person or via Zoom for data collection visits. Several strategies were put in place to minimise study attrition and maximise survey completion. These included reminding both schools and students about upcoming data collection sessions, having the team provide an overview of the Future Proofing Study and deliver a brief presentation about the value of participating in research at each data collection session, offering snacks for the students to enjoy at the data collection visits and enabling students who were away from school or who moved school the option to complete surveys in their own time. Additionally, at 6 months post baseline, participants were sent a text message and instructed to complete the primary outcome measure independently, in their own time.

#### Primary outcome

The Patient Health Questionnaire-Adolescent Version (PHQ-A; 20) is a nine-item questionnaire assessing depressive symptoms in the preceding 2 weeks. It has high specificity (94%) and sensitivity (73%) for major depressive disorder.^17 18^ Internal consistency was α=0.88 baseline; α=0.89 endpoint.

#### Secondary outcomes

Secondary outcomes were anxiety symptoms,^19 20^ psychological distress^21 22^ and insomnia symptoms.^23 24^ See online supplemental file 1 for full descriptions.

#### Programme engagement and acceptability

Engagement was measured by assessing the number of SPARX modules completed, and an analysis evaluating engagement level on the primary outcome was planned.^9^ Acceptability was also measured at the 6-week post baseline assessment, in the intervention group only. Guided by previous studies,^11^ participants were categorised into high (completed≥4 modules), low (completed 1–3 modules) or no (completed 0 modules) engagement groups. Acceptability was measured using an instrument designed specifically for SPARX^11^ with 11 items assessing barriers to use, programme usefulness, how easy or difficult the programme was to understand, behaviour change and whether participants would recommend the programme to others.

### Sample size

Separate sample size estimates were calculated for each stage of the study. For stage 1, 1244 students were needed to detect a 0.30 mean standardised difference between conditions, with 80% power and an α value of 0.05 (two tailed). A correlation of 0.5 between baseline and endpoint symptom scores was assumed. A design effect was calculated based on an intraclass correlation coefficient of 0.03 and a mean cluster size of 50 students, yielding an effect of 2.47. This estimate allowed for 30% attrition at the primary endpoint. Calculations were informed by our previous trial of SPARX.^11^

### Study conditions

#### Intervention

SPARX is a web-based cognitive–behavioural treatment for adolescent depression.^10^ SPARX was adapted into a prevention programme and evaluated in schools confirming effects on depression (d=0.29; 11). An app version was created for use in the current study. Core skills covered include emotion identification, emotion regulation, behavioural activation, challenging unhelpful thoughts and problem-solving. SPARX is delivered across seven 20 min modules in a game format where participants navigate through a fantasy world. Participants were encouraged to do one-to-two modules per week without human guidance and had access to SPARX for 6 weeks on their smartphones. Most intervention schools scheduled a long in-class session for the baseline assessment, immediately after which students were instructed to download SPARX and begin the first module. Schools were instructed to provide some class time for completion of the subsequent intervention modules. Specifically, schools were required to schedule a minimum of 4×20 min in class sessions for students to complete the first four modules of SPARX, with the remaining modules to be completed in students’ own time or further class time if provided by the school.

#### Control

Participants in the control group did not have access to any programme during the 6-week intervention period.

Participants in both groups were invited to use a data collection app during the 6 weeks between baseline and post intervention, which included cognitive tasks and passively collected sensor data.^9^ This was not part of the trial and will be published separately.

### Statistical methods

The statistical approach was predetermined.^25^ Analyses were conducted in STATA (V.18). Analysis of the primary and secondary outcomes used an intention-to-treat approach, including all participants regardless of intervention received. The significance of the primary outcome was based on a planned contrast comparing change in depressive symptoms from baseline to 12 months between the trial arms using a mixed effects model repeated measure analysis (MMRM). Secondary outcomes also used this method. Additional analyses were undertaken to examine the level of engagement with the intervention (high vs low vs no vs control) as a between-group factor. Acceptability data were analysed descriptively. Subgroup analyses were conducted to examine whether there were effects for groups based on gender (female/male) and probable depression caseness at baseline (PHQ-A≥15, yes/no).

Missing outcome data were assumed to be missing at random. A random intercept for school accommodated potential clustering effects, and estimates of the intracluster correlation coefficients are reported. An unconstrained variance–covariance matrix was used to model within-individual dependencies. Df were estimated using the Kenward-Roger method with tests having more than 1000 df reported as z tests.

### Findings

**Figure 1:**
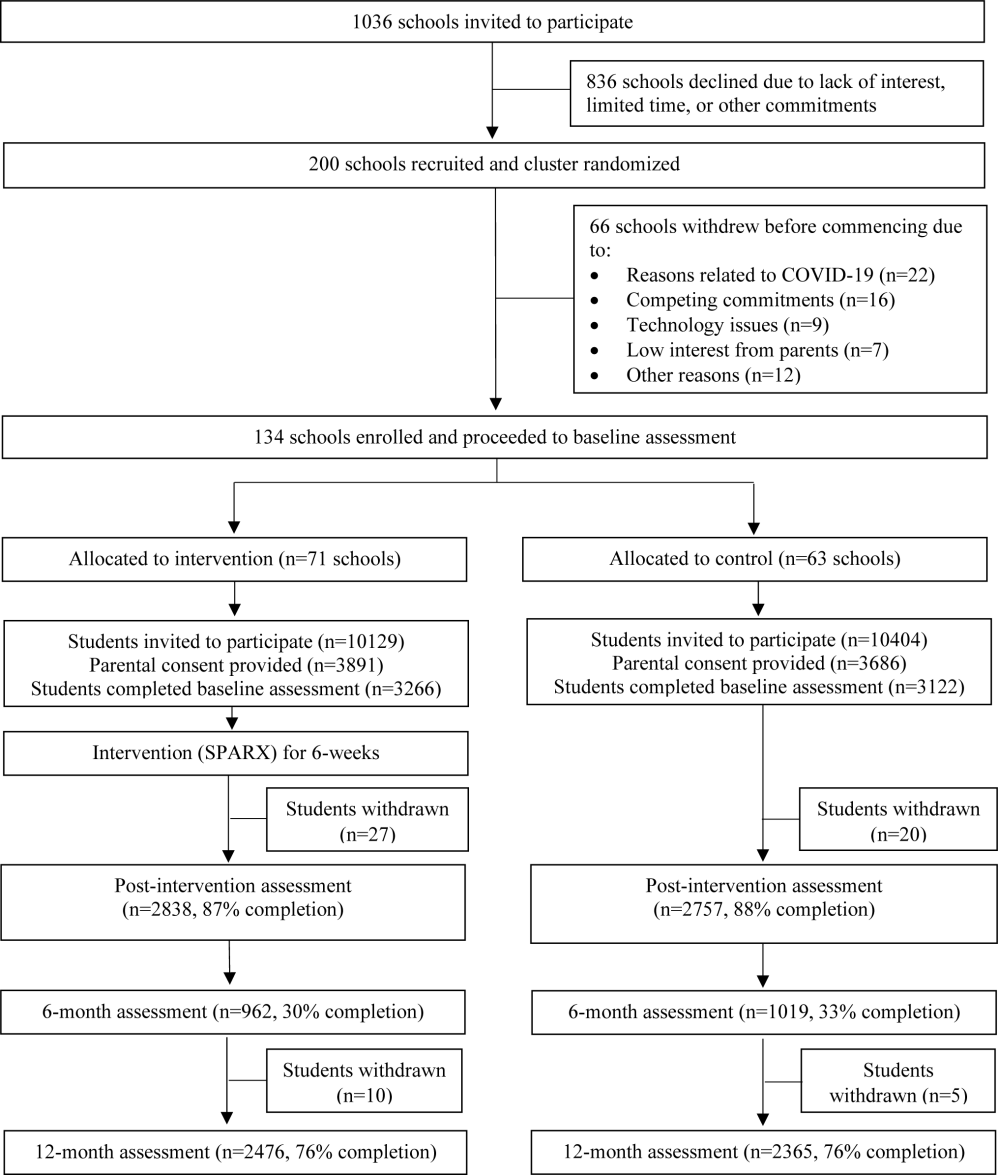
Study flow.

See figure 1 for study flow. A total of 200 schools consented to participate. However, baseline data collection coincided with multiple COVID‐19 lockdowns, and 66 schools withdrew from the study prior to baseline (see online supplemental file 2 for reasons). There were 134 schools allocated as clusters in the final sample, 77 (57.5%) of which were government schools and 57 (42.5%) non‐government schools. From these 134 schools, 20 533 Year 8 students were invited to participate. Consent was obtained from 7577 parents and baseline data collected from 6388 students (consent rate 31%). There were 3266 students (71 schools) randomised to the intervention group, and 3122 students (63 schools) randomised to the control group. Although schools were required to provide in-class time for intervention completion, many schools reported time constraints and school-level restrictions on in-class phone use as barriers. Therefore, schools reported that in these instances, they instructed students to complete SPARX in their own time, outside of school, with regular reminders provided by school staff. Follow-up data at the primary endpoint were provided by 4841 participants (24.2% attrition), with 62 students (0.97%) withdrawing during the study. Primary outcomes were assessed 12 months after baseline (mean=55.5 weeks). The intraclass correlation coefficient for schools at the primary endpoint was 0.051, 95% CI: 0.035 to 0.068.

### Participant characteristics

See table 1 for baseline characteristics.

**Table 1:** Baseline characteristics of study participants and schools by group

	Intervention	Control
	n=3266	n=3122
**Participant-level characteristics**		
Mean age (SD)	13.9 (0.55)	13.9 (0.59)
Gender identity, n (%)		
Female	1621 (49.6)	1501 (48.1)
Male	1494 (45.7)	1478 (47.3)
Non-binary	64 (2.0)	53 (1.7)
Other	27 (0.8)	41 (1.3)
Prefer not to say	60 (1.8)	49 (1.6)
Aboriginal and/or Torres Strait Islander identity, n (%)		
Not Aboriginal and/or Torres Strait Islander	2998 (91.8)	2901 (92.9)
Aboriginal and/or Torres Strait Islander	182 (5.6)	156 (5.0)
Prefer not to say	86 (2.6)	65 (2.1)
Country of birth n (%)		
Australia	2983 (91.3)	2859 (91.6)
Others	283 (8.7)	263 (8.4)
Language spoken most at home, n (%)		
English	3030 (92.8)	2952 (94.6)
Others	235 (7.2)	170 (5.4)
Clinical symptoms, mean (SD)		
Depression (PHQ-9)	7.33 (6.21)	7.39 (6.39)
Anxiety (CAS-8)	8.28 (5.49)	8.23 (5.56)
Distress (DQ5)	11.26 (5.06)	11.42 (5.08)
Insomnia (ISI)	7.05 (5.56)	7.08 (5.53)
**School-level characteristics**		
School state, n (%)		
New South Wales	2930 (89.7)	2562 (82.1)
Victoria	91 (2.8)	320 (10.2)
Queensland	17 (0.5)	196 (6.3)
Western Australia	161 (4.9)	22 (0.7)
South Australia	67 (2.1)	22 (0.7)
School location, n (%)		
Metropolitan	2424 (74.2)	2431 (77.9)
Regional	842 (25.8)	691 (22.1)
School sector, n (%)		
Government school	1834 (56.2)	1418 (45.4)
Non-government school	1432 (43.8)	1704 (54.6)
School type, n (%)		
Coeducational	2570 (78.7)	2024 (64.8)
Gender selective	696 (21.3)	1098 (35.2)
School size, mean (SD)		
FTE students	863.2 (268.7)	1050 (254.0)
FTE teaching staff	69.8 (25.0)	83.1 (23.6)
School ICSEA, median (SD)	1041 (88)	1055 (70)

CAS-8, Children’s Anxiety Scale Short Form-8 (Anxiety); DQ5, Distress Questionnaire 5 (Psychological Distress); FTE, full-time equivalent; ICSEA, Index of Community Socio-Educational Advantage; ISI, Insomnia Severity Index; PHQ-A, Patient Health Questionnaire for Adolescents (Depression).

### Primary outcome

Estimated marginal means and between-group and within-group effect sizes are presented in table 2. Intention-to-treat analyses showed no significant differences in change in depressive symptoms between the intervention and control group from baseline to post intervention (mean change difference= −0.02, z= −0.18, 95% CI: −0.26 to .21, p=0.86), a borderline change from baseline to 6 months, with the intervention group showing a decrease of 0.36 units greater than the control group (z= −1.94, 95% CI: −0.73 to 0.00, p=0.05) and no significant between-group changes from baseline to 12-month primary endpoint (mean change difference= −0.05, z= −0.32, 95% CI: −0.36 to 0.23, p=0.75). Notably, the response rate at 6 months when questionnaires were administered out of class was only 31% and so this result needs to be interpreted cautiously and is unlikely to reflect the participant experiences of the broader sample.

**Table 2:** Estimated marginal means, SE and within-group effect sizes for depression, anxiety, distress and insomnia symptoms

Measure	Group	Baseline		Post intervention		6 months		12 months primary endpoint		Baseline to post within group ES		Baseline to 12 months within group ES	
		EMM	SE	EMM	SE	EMM	SE	EMM	SE	SMD	(95% CI)	SMD	(95% CI)
PHQ-A	Intervention	7.39	0.20	6.96	0.20	6.20	0.22	7.29	0.20	0.06	0.04 to 0.09	0.02	−0.02 to 0.05
	Control	7.72	0.21	7.31	0.21	6.89	0.22	7.67	0.21	0.06	0.04 to 0.09	0.01	−0.03 to 0.04
CAS-8	Intervention	8.33	0.19	7.81	0.20	–	–	7.72	0.19	0.11	0.09 to 0.14	0.09	0.06 to 0.13
	Control	8.48	0.20	8.04	0.21	–	–	7.71	0.20	0.14	0.11 to 0.16	0.08	0.05 to 0.11
DQ5	Intervention	11.30	0.18	10.95	0.18	–	–	11.30	0.18	0.07	0.04 to 0.10	−0.00	−0.00 to 0.03
	Control	11.69	0.19	11.17	0.19	–	–	11.82	0.19	0.20	0.07 to 0.13	−0.03	−0.06 to 0.01
ISI	Intervention	7.11	0.17	7.06	0.16	–	–	7.13	0.17	0.01	−0.02 to 0.04	−0.01	−0.04 to 0.03
	Control	7.30	0.16	7.18	0.17	–	–	7.32	0.17	0.02	−0.01 to 0.05	−0.00	−0.04 to 0.03

Note that only the PHQ-A was administered at 6 months.

CAS-8, Spence Child Anxiety Scale-Short Form; DQ5, Distress Questionnaire 5; EMM, estimated marginal means; ES, effect size; ISI, Insomnia Severity Index; PHQ-A, Patient Health Questionnaire—Adolescent Version; SMD, standardised mean difference.

Within-group contrasts showed a significant reduction in depressive symptoms from baseline to post intervention, with a decrease of −0.43 units in the intervention group (z= −5.11, 95% CI: −0.59 to −0.26, p<0.001), and −0.41 units in the control group (z= −4.79, 95% CI: −0.57 to −0.24, p<0.001). From post intervention to 6 months, symptoms continued to reduce in both the intervention (mean change= −0.76, z= −5.94, 95% CI: −1.01 to −0.51, p<0.001), and control group (mean change= −0.42, z= −3.36, 95% CI: −0.67 to −0.18, p<0.001), and then significantly increased from 6 months to 12 months (intervention, mean change=1.09, z=8.23, 95% CI: 0.83 to 1.35, p<0.001; control, mean change=0.77, z=5.98, 95% CI: 0.52 to 1.03, p<0.001), but not differentially so (*t*(172)= −1.28, 95% CI: −0.96 to 0.20, p=0.20). A contrast comparing symptoms from baseline to 12 months showed no significant change in the intervention (mean change= −0.10, z= −0.92, 95% CI: −0.32 to 0.12, p=0.36), nor control group (mean change= −0.05, z= −0.47, 95% CI: −0.27 to 0.17, p=0.64).

### Secondary outcomes

See table 2 for full details. There were no significant differences in change in anxiety symptoms between groups from baseline to post intervention (mean change difference= −0.16, z= −0.70, 95% CI: −0.35 to 0.16, p=0.49), nor to 12 months (mean change difference= −0.09, z=1.56, 95% CI: −0.04 to 0.35, p=0.12). Results for psychological distress were similar, with no between-group differences in change scores from baseline to post intervention (mean change difference=0.16, z=1.69, 95% CI: −0.03 to 0.35, p=0.09), nor to 12 months (mean change difference= −0.13, z= −1.02, 95% CI: −0.38 to 0.12, p=0.31). For insomnia, there were no between-group differences in change between baseline and post intervention (mean change difference=0.07, z=0.64, 95% CI: −0.14 to 0.28, p=0.52), nor at 12 months (mean change difference=0.00, z=0.00, 95% CI: −0.28 to 0.28, p=0.99). Within-group results are reported in online supplemental file 3.

### Subgroup analyses

Analyses of the PHQ-A were repeated separately for males and females, and for participants who met criteria for probable depression caseness (PHQ-A≥15) at baseline. For males, mean scores fell by 0.53 points more in the intervention group than the control group from baseline to 6 months (95% CI: 0.01 to 1.07, z= −1.98, p=0.05). No other differences were observed.

### Intervention engagement

Of participants allocated to SPARX, 87% installed the app, 57% completed the first module and 12% completed all seven modules. 43% of participants comprised the ‘no engagement’ group (zero modules completed), 35% were in the ‘low engagement’ group (1–3 modules) and 22% in the ‘high engagement’ group (4+ modules). Females were more likely to be high engagers compared with males (OR=1.26, 95% CI: 1.03 to 1.54), as were those with no previous diagnosis of a mental health condition (OR=1.37, 95% CI: 1.07 to 1.74).

An MMRM was conducted using these engagement categories and the control group as a between-group factor. There were no differences in depression symptom change between the intervention and the control group, nor between the no and low engagement groups at any of the assessment points (all p values>0.05). A contrast comparing the high engagement group with the control group showed a significant difference in depression symptom change from baseline to post intervention (*t*(342.16)*=* −0.52; 95% CI: 0.14 to 0.89, p<0.01). However, symptom differences at baseline between these groups preclude firm conclusions being drawn from this finding, *t*(349.756)= −1.97; CI: −1.40 to 0.00, p*<*0.05. See online supplemental file 4 for full details.

### Intervention acceptability

Of participants who accessed SPARX, approximately half (55.8%) reported finding SPARX ‘useful’ or ‘very useful’, and 50.9% of participants indicated they would use this kind of programme again in the future. Just over half (52.9%) of participants found the programme easy to understand, while 13.1% found it difficult to understand, with 34% finding it neither difficult nor easy to understand.

## Discussion

The hypotheses that students receiving the SPARX intervention would report lower symptoms of depression, anxiety, distress and insomnia relative to the control group at the 12-month end point were not supported. These findings contrast with meta-analyses that have reported small preventive effects of depression and anxiety for school-based programmes.^4 5^

Participant engagement with SPARX was low. 43% of the sample did not engage with the intervention at all, while only 22% of participants completed at least four of seven modules, a completion rate which has been identified as necessary for prevention.^11^ Despite SPARX being aligned with adolescents’ preferences for self-directed, gamified approaches,^26^ students were expected to complete the programme in their own time, with minimal, if any, accountability. Results from this and another previous school-based mindfulness study^7^ indicate that young people are unlikely to engage with self-directed mental health activities or practice in their own time, with half of the students not completing the required home practice exercises.^27^ This is comparable to the 43% of students in the current trial who were ‘non-engagers’. The null results of the current trial may be related in part to the reliance on young people to engage with the intervention themselves on occasions when schools did not schedule class time. To this point, delivery in the school context added a layer of complexity, with most schools unable to allocate time for in-class SPARX delivery. This differs from our previous SPARX trial where the intervention was delivered in class time to school students aged 16–17 years.^11^ In our previous study, schools were required to share the class timetable and scheduling of intervention sessions, which was verified by the research team. This delivery model (where all schools allocated time for intervention completion) led to an SPARX completion rate of 59%. Conversely, in the current study, schools were provided with greater autonomy and less researcher involvement in scheduling to emulate a more realistic implementation scenario outside of a research trial. Results showed that this model led to many schools failing to allocate in-class time for intervention completion, with an SPARX completion rate of 22%. This points to the importance of schools allocating class time as being necessary for intervention completion.

The acceptability level of SPARX also warrants consideration. For participants who did the programme, only half reported that it was useful, suggesting that for many, it was not relevant to their circumstances or experiences. Using an adapted treatment programme and framing SPARX as explicitly mental health focused may have also been an engagement barrier, as some students may have felt the programme was not relevant to them. Acceptability is likely to have been impacted by the programme look and feel, which was developed >10 years ago, with now-dated graphics and gaming features, an aspect supported by participant feedback (eg, “I could be playing way better games”). Another related possibility is that students aged 13–14 years were not developmentally ready for the content, consistent with studies testing the intervention in slightly older adolescents.^10 11^ A Year 8 sample aged 13–14 years was selected as the target group on the basis that there is a rapid increase in depressive symptoms from this age.^12^ Although this age range aligns with the delivery of other school-based cognitive–behavioural therapy programmes,^4 28^ it is possible that some students may have found the SPARX content too advanced. This is supported by the data, with only about half of participants reporting that the material was easy to understand. Therefore, SPARX might be better suited to an older age group. Future studies involving SPARX should pilot test the intervention in the target age group and introduce tailoring to ensure developmental appropriateness if necessary.

It is also important to consider the timing of this study, which took place during the COVID-19 pandemic. The pandemic had a significant impact on young people’s lives,^29^ and perhaps it is unrealistic to expect a brief, self-directed, low-intensity intervention to have an effect in the context of a global event of such magnitude.

Notably, an unexpected pattern of results from this study was that mental health symptoms did not increase from baseline to 12 months. Large-scale, nationally representative adolescent data show that disorder symptoms increase annually at a population level.^30^ We speculate that the strength of the study activities in both control and intervention groups may have contributed to the lack of symptom increase over time. This included three visits from our research team for data collection purposes and also involved the presentation of information about the work of our mental health organisation, the Black Dog Institute and a presentation about the value of participating in mental health research. It is possible that these visits were valuable and beneficial to students in both arms, which may have contributed to the lack of symptom increase typically detected at this age.

The current findings suggest caution in the delivery of universal interventions for the prevention of depression in schools. Our study joins several recent high-quality studies investigating a mindfulness^7^ and cognitive–behavioural programme,^8^ which both found no effect on depression at 12–18 months follow-up. However, before concluding that programmes like these should not be routinely delivered, nuance is needed in the consideration of the evidence, including the implementation of the programme. Firm conclusions about the effectiveness of this intervention are complicated by the low levels of SPARX completion and the associated implementation model. This study advances the field by establishing that schools require significant support to schedule in-class mental health activities, which need to be balanced with other school priorities. This study also contributes to the evidence base showing that students are unlikely to complete mental health activities in their own time. Future studies could involve greater consideration about the type of intervention being delivered, when and how it is administered and what developmental stage it is appropriate for. Incorporating student perspectives to guide the development and delivery of these interventions is also critical, and ensuring this engagement is ongoing will be a priority. Finally, it may be unrealistic to expect that brief psychological interventions, typically delivered individually to students only once, will have long-term meaningful effects.

The strengths of this study include the size and representative sample, attention to implementation factors and an intervention with an evidence base. Limitations include the low consent rate to participate, the failure of many schools to schedule in-class time for intervention completion, low student engagement with the intervention and the influence of the COVID-19 pandemic. It would be beneficial for future school-based studies to undertake careful pilot and feasibility testing of the intervention and associated implementation framework across a range of diverse schools, in order to provide valuable information about the feasibility of the delivery method and likely intervention engagement levels.

This study showed that a cognitive–behavioural programme, when implemented at scale to school students, did not improve mental health relative to a control group, 12 months later. Most participants did not engage sufficiently with the intervention, and most likely did not receive the necessary exposure required for preventive effects.

### Clinical implications

Implementation factors need to be considered in the context of delivering universal digital mental health programmes in schools. School-based mental health policy should be evidence-informed and future research could evaluate implementation models that require schools to (1) allocate time in-class for intervention completion, (2) deliver programmes that target specific risk factors and (3) focus on areas most relevant to young people in the school context.

## Supplementary material

10.1136/bmjment-2024-301426 online supplemental file 1

## Data Availability

Data are available on reasonable request.

## References

[1] Ebert DD, Cuijpers P (2018). It Is Time to Invest in the Prevention of Depression. JAMA Netw Open.

[2] Klaufus L, Verlinden E, van der Wal M (2022). Adolescent anxiety and depression: burden of disease study in 53,894 secondary school pupils in the Netherlands. BMC Psychiatry.

[3] Solmi M, Radua J, Olivola M (2022). Age at onset of mental disorders worldwide: large-scale meta-analysis of 192 epidemiological studies. Mol Psychiatry.

[4] Werner-Seidler A, Spanos S, Calear AL (2021). School-based depression and anxiety prevention programs: An updated systematic review and meta-analysis. Clin Psychol Rev.

[5] Stockings EA, Degenhardt L, Dobbins T (2016). Preventing depression and anxiety in young people: a review of the joint efficacy of universal, selective and indicated prevention. Psychol Med.

[6] Araya R, Fritsch R, Spears M (2013). School intervention to improve mental health of students in Santiago, Chile: a randomized clinical trial. JAMA Pediatr.

[7] Kuyken W, Ball S, Crane C (2022). Effectiveness and cost-effectiveness of universal school-based mindfulness training compared with normal school provision in reducing risk of mental health problems and promoting well-being in adolescence: the MYRIAD cluster randomised controlled trial. Evid Based Ment Health.

[8] Andrews JL, Birrell L, Chapman C (2023). Evaluating the effectiveness of a universal eHealth school-based prevention programme for depression and anxiety, and the moderating role of friendship network characteristics. Psychol Med.

[9] Werner-Seidler A, Huckvale K, Larsen ME (2020). A trial protocol for the effectiveness of digital interventions for preventing depression in adolescents: The Future Proofing Study. Trials.

[10] Merry SN, Stasiak K, Shepherd M (2012). The effectiveness of SPARX, a computerised self help intervention for adolescents seeking help for depression: randomised controlled non-inferiority trial. BMJ.

[11] Perry Y, Werner-Seidler A, Calear A (2017). Preventing Depression in Final Year Secondary Students: School-Based Randomized Controlled Trial. J Med Internet Res.

[12] Kwong ASF, Manley D, Timpson NJ (2019). Identifying critical points of trajectories of depressive symptoms from childhood to young adulthood. J Youth Adolesc.

[13] Campbell MK, Piaggio G, Elbourne DR (2012). Consort 2010 statement: extension to cluster randomised trials. BMJ.

[14] Beames JR, Werner-Seidler A, Hodgins M (2023). Implementing a Digital Depression Prevention Program in Australian Secondary Schools: Cross-Sectional Qualitative Study. JMIR Pediatr Parent.

[15] Werner-Seidler A, Maston K, Calear AL (2023). The Future Proofing Study: Design, methods and baseline characteristics of a prospective cohort study of the mental health of Australian adolescents. Int J Methods Psychiatr Res.

[16] ACARA (2020). Guide to understanding the Index of Community Socio-Educational Advantage (ICSEA).

[17] Johnson JG, Harris ES, Spitzer RL (2002). The patient health questionnaire for adolescents: validation of an instrument for the assessment of mental disorders among adolescent primary care patients. J Adolesc Health.

[18] Kroenke K, Spitzer RL, Williams JB (2001). The PHQ-9: validity of a brief depression severity measure. J Gen Intern Med.

[19] Spence SH, Barrett PM, Turner CM (2003). Psychometric properties of the Spence Children’s Anxiety Scale with young adolescents. J Anxiety Disord.

[20] Spence SH (1998). A measure of anxiety symptoms among children. Behav Res Ther.

[21] Batterham PJ, Sunderland M, Carragher N (2016). The Distress Questionnaire-5: Population screener for psychological distress was more accurate than the K6/K10. J Clin Epidemiol.

[22] Batterham PJ, Werner-Seidler A, O’Dea B (2024). Psychometric properties of the Distress Questionnaire-5 (DQ5) for measuring psychological distress in adolescents. J Psychiatr Res.

[23] Bastien CH, Vallières A, Morin CM (2001). Validation of the Insomnia Severity Index as an outcome measure for insomnia research. Sleep Med.

[24] Chung KF, Kan K-K, Yeung W-F (2011). Assessing insomnia in adolescents: comparison of Insomnia Severity Index, Athens Insomnia Scale and Sleep Quality Index. Sleep Med.

[25] Werner-Seidler A, Huckvale K, Larsen ME (2020). A trial protocol for the effectiveness of digital interventions for preventing depression in adolescents: The Future Proofing Study. Trials.

[26] Kenny R, Dooley B, Fitzgerald A (2016). Developing mental health mobile apps: Exploring adolescents’ perspectives. Health Informatics J.

[27] Montero-Marin J, Hinze V, Crane C (2023). Do Adolescents Like School-Based Mindfulness Training? Predictors of Mindfulness Practice and Responsiveness in the MYRIAD Trial. J Am Acad Child Adolesc Psychiatry.

[28] Teesson M, Newton NC, Slade T (2020). Combined prevention for substance use, depression, and anxiety in adolescence: a cluster-randomised controlled trial of a digital online intervention. Lancet Digit Health.

[29] Racine N, McArthur BA, Cooke JE (2021). Global Prevalence of Depressive and Anxiety Symptoms in Children and Adolescents During COVID-19: A Meta-analysis. JAMA Pediatr.

[30] Mojtabai R, Olfson M, Han B (2016). National Trends in the Prevalence and Treatment of Depression in Adolescents and Young Adults. Pediatrics.

