# Supplementary material for: Future Proofing Study: a cluster randomised controlled trial evaluating the effectiveness of a universal school-based cognitive–behavioural programme for adolescent depression
Source: BMJ Ment Health. 2025 Mar 14;28(1):e301426. doi: 10.1136/bmjment-2024-301426 (PMC11927416; doi:10.1136/bmjment-2024-301426)
Supplement: online supplemental file 1 [file bmjment-28-1-s001.docx]

Supplementary Materials

Contents

1. Supplemental File 1. Secondary outcome measures
2. Supplemental File 2. Reasons for school withdrawal
3. Supplemental File 3. Secondary outcomes – within-group comparison statistics
4. Supplemental File 4. Engagement with SPARX analysis

Supplemental File 1

Secondary outcome measures

**Anxiety.** Anxiety was measured using the Children's Anxiety Scale-Short Form CAS-8, an eight‐item measure of anxiety, based on the Spence Children's Anxiety Scale [1]. The CAS‐8 includes assessment of generalized anxiety and social anxiety and has good reliability and provides population‐level, standardized norms, with a range of 0–24 [higher score indicates greater anxiety; 1,2]. The internal consistency of the CAS‐8 was high in the current study (α = 0.88).

**Psychological Distress.** Psychological distress was measured using the Distress Questionnaire-5 [3], a five-item tool with strong psychometric properties in adolescent school samples [4]. Scores range from 5–25 (higher score indicates greater distress). The internal consistency of the DQ5 in the current study was good (α = 0.88).

**Insomnia**. Insomnia symptoms were measured using the Insomnia Severity Index (ISI), a psychometrically sound, seven-item self-report measure of insomnia symptoms over the previous two weeks [5]. Scores range from 0–28 with a higher score indicating higher levels of insomnia. The ISI has been validated in adolescent samples [6]. The internal consistency of insomnia symptoms in the current study was high (α = 0.86).

1. Spence SH, Barrett PM, Turner CM. Psychometric properties of the Spence Children's Anxiety Scale with young adolescents. Journal of Anxiety Disorders. 2003;17(6):605-25.
2. Spence SH. A measure of anxiety symptoms among children. Behaviour Research and Therapy. 1998;36(5):545-66.
3. Batterham PJ, Sunderland M, Carragher N, et al. The Distress Questionnaire-5: Population screener for psychological distress was more accurate than the K6/K10. Journal of Clinical Epidemiology. 2016;71:35-42. doi:10.1016/j.jclinepi.2015.10.005.
4. Batterham PJ, Werner-Seidler A, O'Dea B, et al. Psychometric properties of the Distress Questionnaire-5 (DQ5) for measuring psychological distress in adolescents. Journal of Psychiatric Research. 2024;169:58-63. <https://doi.org/10.1016/j.jpsychires.2023.11.004>.
5. Bastien CH, Vallières A, Morin CM. Validation of the Insomnia Severity Index as an outcome measure for insomnia research. Sleep Medicine. 2001;2(4):297-307. <https://doi.org/10.1016/S1389-9457(00)00065-4>.
6. Chung KF, Kan KK-K, Yeung W-F. Assessing insomnia in adolescents: Comparison of insomnia severity index, Athens Insomnia Scale and sleep quality index. Sleep Medicine. 2011;12(5):463-470. doi: 10.1016/j.sleep.2010.09.019.

Supplemental File 2

Reasons for school withdrawal from the study

| **Reason for school withdrawal** | | **Intervention** | **Control** |
| --- | --- | --- | --- |
| Competing commitments | 9 | | 7 |
| COVID | 9 | | 13 |
| Low interest from parents | 3 | | 4 |
| Technology | 5 | | 4 |
| Other | 4 | | 8 |
| Total | 30 | | 36 |

Supplemental File 3

Secondary outcomes – within group comparison statistics

For anxiety symptoms, there were within-group reductions of 0.6 units in symptoms from baseline to post-intervention in the intervention group [z=-8.73, 95%CI: -.75 to -.47, p<.001] and .76 units in the control group [z=-10.79, 95%CI: -.26 to .-90, p=.<.001]. From post-intervention to 12-months, the control group showed continued reduction in symptoms by .33 units [z=3.68 95%CI: .16 to .51, p<.001], while the intervention group showed no change from post to 12-months, [mean change=.09, z=.96, 95%CI: -.09 to .26, p=.33]. Mean symptoms at 12-months showed a significant reduction of .53 symptom units from baseline in the intervention group, [z=-5.69, 95%CI: -.71 to -.34, p<.001], and a reduction of .43 units in the control group, [z=-4.65, 95%CI: -.61 to -.25, p<.001].

For psychological distress, within-group contrasts showed a significant reduction in symptoms from baseline to post-intervention with a magnitude of -.35 units in the intervention group [*z*=-5.25, 95%CI: -.48 to -.22, *p*<.001] and -.51 units in the control group [*z*=-7.54, 95%CI: -.65 to -.38, *p*<.001], which then increased between post-intervention and 12-months, [intervention, mean change =.35, *z*=4.04, 95%CI: 18 to .53, *p*<.001; control, mean change=.65, *z*=7.30, 95%CI: .47 to .82, *p*<.001] such that there was no overall symptom change from baseline to 12-months in the intervention, [mean change=.00, *z*=0.02, 95%CI: -.17 to .18, *p*=.99], nor control arm, [mean change=.13, *z*=1.46, 95%CI: -.05 to .31, *p*=.15].

For insomnia symptoms, there were no significant within-group symptom changes from baseline to post-intervention, nor baseline to 12-months (all *p*s>.05).

Supplemental File 4

Engagement with SPARX analysis

A contrast comparing the high engagement group with the control group showed a significant difference in depression change from baseline to post-intervention, by 0.18 points (95%CI:.14 to.89, *p*<.01). Interpretation of this differential change is complicated by symptom differences at baseline. However, this effect was due to convergence of the control group from a significantly higher mean at baseline to a lower value post-intervention that was not significantly different from the high engagers. See Figure below. There was no difference in change scores at the 6- or 12-month follow-up time points between the high engagement group and control group (all *p*s>.05). This pattern of results was similar for a contrast comparing change in the high and no engagement group, with a difference in change from baseline to post-intervention, 95%CI: .34 to 1.19, *p*<.001, but no difference in score at this time point (*p*>.05), and again was not found at 6 or 12 months (*p*s>.05).
